# Evaluation of Luffa Rootstocks to Improve Resistance in Bitter Gourd (*Momordica charantia* L.) Against Fusarium Wilt

**DOI:** 10.3390/plants14081168

**Published:** 2025-04-09

**Authors:** Ahmed Namisy, Shu-Yun Chen, Benjapon Sritongkam, Jintana Unartngam, Chinnapan Thanarut, Wen-Hsin Chung

**Affiliations:** 1 Department of Plant Pathology, National Chung Hsing University, Taichung 402, Taiwan; 2 Department of Agronomy, National Chung Hsing University, Taichung 402, Taiwan; 3 Department of Horticulture, Faculty of Agriculture, Ubon Ratchathani University, Ubon Ratchathani 34190, Thailand; benjapon.s@ubu.ac.th; 4 Department of Plant Pathology, Faculty of Agriculture, Kasetsart University, Kamphaeng Saen Campus, Kamphaeng Saen 10900, Thailand; agrjne@ku.ac.th; 5 Division of Pomology, Faculty of Agriculture Production, Maejo University, Chiang Mai 50290, Thailand; 6 Master Program for Plant Medicine and Agricultural Practice, National Chung Hsing University, Taichung 402, Taiwan

**Keywords:** bitter gourd, *Fusarium oxysporum*, cucurbits grafting, resistant rootstock, soil-borne disease

## Abstract

Fusarium wilt in bitter gourd caused by *Fusarium oxysporum* f. sp. *momordicae* (Fomo) is a severe plant disease that affects the world’s bitter gourd (*Momordica charantia* L.) cultivation. This study evaluated nine luffa hybrids for their performance as rootstocks with bitter gourd to control *Fusarium oxysporum* f. sp. *luffae* (Folu) isolate Fomh16 and Fomo isolate Fomo33. In the first evaluation, five hybrids (LF1, LF2, LF3, LF15, and LF16) exhibited resistance to the Fomh16 isolate and showed no symptoms. One hybrid, LF10, was resistant with a mean disease rating (MDR) of 0.9 at 28 days post-inoculation (dpi). Seven luff hybrids that displayed resistant and moderate resistance in the first evaluation were used as rootstocks with susceptible bitter gourd cultivars. Five rootstocks exhibited high resistance to Fomh16 and Fomo33 isolates, with their MDR ranging from 0.0 to 0.7. In addition, the findings revealed that both isolates could colonize the vascular bundle of all resistant luffa rootstocks at 28 dpi. However, the Fomo33 isolate could extend and colonize the vascular bundle of bitter gourd scion when grafted only with rootstock LF5 and LF11. The quantitative PCR results indicated that there were significant differences in the amount of the Fomo33 DNA between the bitter gourd grafted onto LF15 and LF16 rootstocks and the self-grafted plants; however, the pathogen cannot be detected in the bitter gourd scions grafted with resistant rootstocks. These findings provide valuable resistant sources that can be used as rootstocks to manage Fusarium wilt disease in bitter gourd.

## 1. Introduction

Bitter gourd *Momordica charantia* is an important vegetable crop belonging to the family Cucurbitaceae. It is a tropical and subtropical vegetable crop widely cultivated in Asian countries with a cultivated area of about 340,000 ha [1]. Bitter gourd is a highly valued economic crop in Taiwan, with a cultivated area of approximately 1544 hectares. The average yield per hectare is 19,927 kg/ha, as reported in “https://www.afa.gov.tw/eng/ (accessed on 20 July 2023)”. Genus *Momordica* comprises 47 species in Africa and 12 in Asia and Australia [2]. Among the species, three selected species are used as vegetables: *Momordica charantia* L. (Bitter melon), *M. foetida* Schumach (Bitter cucumber), and *M. balsamina* L. (African pumpkin) [3]. In addition to their nutrient importance, bitter gourd fruit also has medicinal benefits [4,5]. However, bitter gourd cultivation faces significant challenges due to Fusarium wilt.

Fusarium wilt in bitter gourd caused by *Fusarium oxysporum* f. sp. *momordicae* (Fomo) is a serious and severe plant disease constraint to bitter gourd (*M. charantia* L.) cultivation in Taiwan [6]. It was reported for the first time in Taiwan by Sun [7]. The pathogen significantly impacts the fruit quality and has become a major limiting factor in producing bitter gourds [8,9], ultimately leading to 100% yield losses in continuously cropped soils [10]. In southeast China, the incidence of the fusarium wilt of bitter gourd is 12.30–56.75% in contaminated fields [11]. The pathogen initially penetrates the root’s epidermis asymptomatically, subsequently colonizing the vascular system; it then produces masses of mycelia and conidia to block water transportation, causing wilting symptoms, followed by leaf yellowing and withering, the darkening and thinning of stem closest to the ground, and finally, the plant death due to disrupting water-conducting xylem vessels [12,13]. Several strategies have been employed to manage Fusarium wilt, including chemical and alternative methods such as solarization, biological control, and resistant cultivars [14,15,16]. However, managing Fusarium wilt diseases is difficult because the pathogen is a soil-borne disease with a broad host range. Breeding for disease-resistant varieties/rootstock is the most economical and effective method to control soil-borne diseases [8,17,18]. However, there are no commercially available resistant bitter gourd varieties in Taiwan.

Despite the challenges of managing Fusarium wilt, grafting with resistant rootstocks is the most effective alternative to controlling soil-borne pathogens worldwide. Vegetable grafting was first introduced in Japan in the late 1920s to control Fusarium wilt in watermelon; this was achieved using resistant rootstocks of Cucurbita species [19,20]. It has been reported that grafting has the potential to enhance resistance or tolerance to different fungal, bacterial, and nematode soil-borne pathogens in solanaceous and cucurbitaceous crops [21,22]. Some primary diseases that can be addressed through grafting include fusarium wilt, bacterial wilt, verticillium wilt, monosporascus root rot, and nematodes [23]. Furthermore, grafted plants enhanced resistance against certain foliar diseases and viruses when susceptible scions are grafted onto specific rootstocks [20,24].

In addition to disease management, the interaction between the scion and rootstock has been reported to improve fruit quality and enhance crop response to various abiotic stresses, including extreme temperatures, salt, drought, and nutrient deficiencies [23,25]. Luffa rootstock has been found to enhance cucumber plants’ salt tolerance significantly, evident from the increased fresh weight, reduced relative electrical conductivity, and improved photochemical efficiency [26]. Also, using luffa as rootstock with cucumber increases water use efficiency, decreases the transpiration rate, and lessens the impact of CO_2_ assimilation capacity in drought stress [27]. These multifaceted advantages make grafting effective for addressing both biotic and abiotic challenges.

Grafting effectively controls Fusarium wilt in cucurbits, including cucumber, melon, and watermelon [15]. The most common rootstocks for cucurbits are bottle gourd, *Cucurbita* spp., luffa (*Luffa cylindrical* (L.) M. Roem.), wax gourd (*Benincasa hispida* (thumb.) Cogn.), and *Citrullus* spp. [20,28]. In Taiwan, luffa is a standard rootstock for bitter gourd and is widely used for summer cultivation because of its resistance to Fusarium wilt and tolerance to heat and flooding [29]. Grafting bitter gourd onto cucurbit rootstocks is a common practice to prevent Fomo. However, the pathogen currently can overcome the resistance and infect the rootstock [6]. Therefore, identifying new sources of resistance to use as rootstock is crucial for controlling Fusarium wilt disease. This research aimed to evaluate the resistance of grafted bitter gourd onto new luffa hybrid rootstocks against both *F. oxysporum* f. sp. *luffae* isolate Fomh16 and *F. oxysporum* f. sp. *momordicae* isolate Fomo33.

## 2. Results

### 2.1. Pathogenicity Test of Fomh16 and Fomo33 Isolates

**Figure 1:**
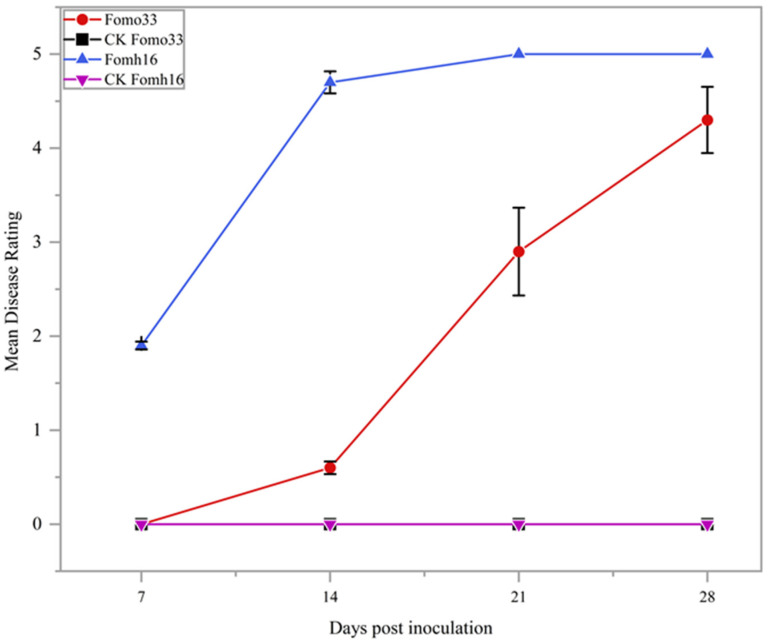
Pathogenicity test of *Fusarium oxysporum* f. sp. *luffae* isolate Fomh16 and *Fusarium oxysporum* f. sp. *momordicae* isolate Fomo33 against the susceptible cultivars (cv. Shimmery) and (cv. Moon shine), respectively, indicated by the mean disease ratings at 7-, 14-, 21-, and 28-days post-inoculation. Error bars represent the standard deviation of triplicates.

The Folu isolate Fomh16 and Fomo isolate Fomo33 were tested for their pathogenicity against two cultivars: luffa (cv. Shimmery) and bitter gourd (cv. Moon shine). The results showed that the Fomh16 isolate was highly pathogenic to the luffa cultivar with MDR 5, and AUDPC was 60.4 during the period of 14 to 21 dpi and not pathogenic to bitter gourd at 28 dpi. On the other hand, the Fomo isolate Fomo33 was highly pathogenic to bitter gourd with MDR 4.3, and AUDPC was 38.3 at 21 dpi but not pathogenic to luffa cultivar at 28 dpi (Figure 1).

### 2.2. Evaluation of Luffa Hybrids to Fusarium Wilt Resistance

Nine F1 hybrids were obtained by crossing four resistant luffa lines. These hybrids were evaluated for their resistance to Folu isolate Fomh16. Among these hybrids, five, LF1, LF2, LF3, LF15, and LF16, showed high resistance and did not exhibit any symptoms at 28 dpi; one hybrid, LF10, was resistant with MDR 0.9 and AUDPC 17.8, while other hybrids, LF5 and LF11, showed moderator resistance with MDR 1.2 and 1.6 and AUDPC 20.5 and 29.0, respectively; and LF6 was moderately susceptible with MDR 2.5 and AUDPC 45.9 (Table 1). The susceptible control *La* (cv. Shimmery) exhibited the expected results, with all plants completely wilting within 14 days after infection dpi (Table 1).

Due to the limited number of seeds, only the luffa hybrids that showed resistant and moderator resistance in the first evaluation LF1, LF2, LF3, LF5, LF15, LF16, and LF11 were used as rootstocks in grafting with a susceptible bitter gourd cultivar. The grafted plants were then evaluated for their resistance against Fomh16 and Fomo33 isolates. Out of seven rootstocks tested for their resistance to the Fomh16 isolate, two rootstocks LF15 and LF16 showed resistant response to the Fomh16 isolate without displaying any symptoms at 28 dpi, three rootstocks, LF1, LF2, and LF11, were also resistant, with their MDR ranging from 0.3 to 0.7 and AUDPC ranging from 4.7 to 11.4. One rootstock, LF5, was moderately resistant with MDR 1.6 and AUDPC 25.9 (Table 2). After inoculating the grafted plants with the Fomo33 isolate, two rootstocks, LF15 and LF16, were resistant and showed no symptoms, whereas three rootstocks, LF1, LF2, and LF3, were resistant, with MDR ranging from 0.3 to 0.5 and AUDPC ranging from 4.9 to 7.9. Two rootstocks, LF5 and LF11, were moderately resistant and susceptible, respectively (Table 2).

**Figure 2:**
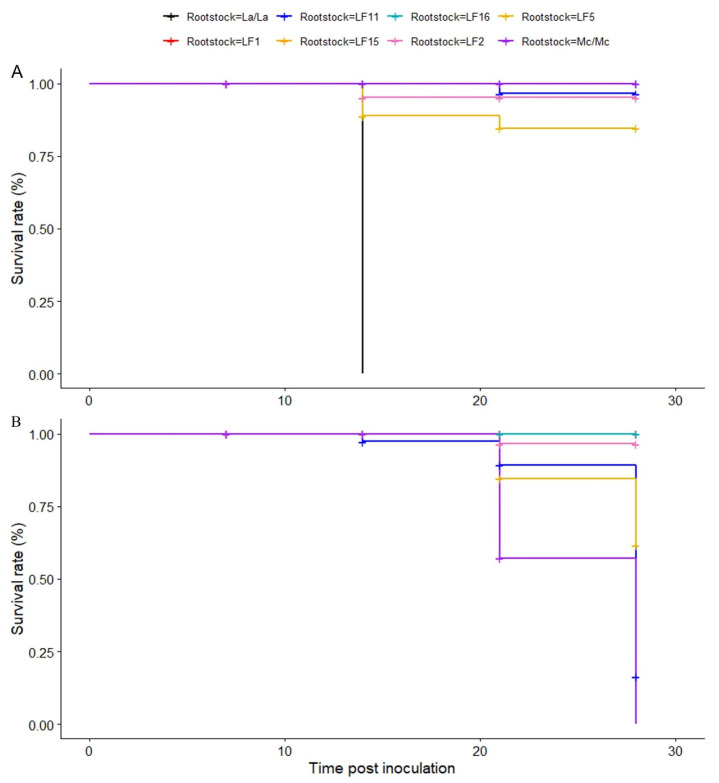
Kaplan–Meier survival curves representing survival rates of bitter gourd plants grafted onto luffa hybrids until 28 dpi. (**A**): grafted plants inoculated with *Fusarium oxysporum* f. sp. *luffae* isolate, Fomh16; (**B**): grafted plants inoculated with *Fusarium oxysporum* f. sp. *momordicae* isolate Fomo33.

The survival rates of grafted plants inoculated with the Fomh16 and Fomo33 isolates varied significantly among different rootstock genotypes. For plants inoculated with the Fomh16 isolate, the rootstocks LF1, LF15 and LF16 achieved a 100% survival rate at 28 dpi. In addition, the rootstocks LF5, LF2, and LF11 displayed survival rates of 84.7%, 95.1%, and 96.5%, respectively, at 28 dpi. As expected, self-grafted luffa *La/La* did not survive beyond 14 dpi, whereas self-grafted bitter gourd *Mc/Mc* maintained a 100% survival rate at 28 dpi (Figure 2). In the case of plants inoculated with the Fomo33 isolate, the rootstocks LF1, LF15, and LF16 also exhibited high survival rates, achieving 100% at 28 dpi, while the LF2 rootstock displayed survival rates of 96.5%. Conversely, the rootstocks LF5 and LF11 showed significantly lower survival rates of 12.7% and 16.2%, respectively, at 28 dpi. The self-grafted luffa *La/La* showed the expected results with a 100% survival rate, while the self-grafted bitter gourd *Mc/Mc* did not survive at 28 dpi (Figure 2).

### 2.3. Colonization of Grafted Plants by Fomh16 and Fomo33 Isolates

**Figure 3:**
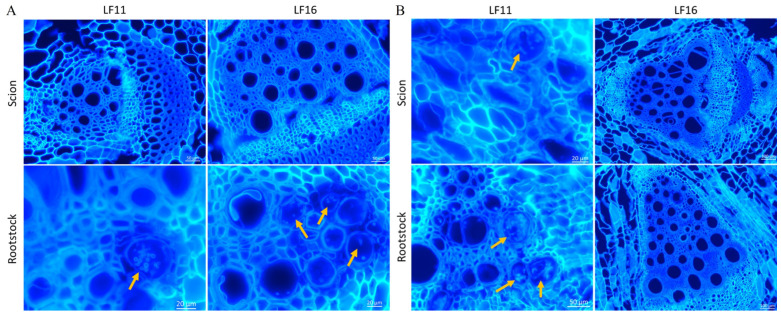
The cross-section in the scion and rootstock of bitter gourd plants grafted onto luffa hybrids LF11 and LF16 at 28 dpi. (**A**): grafted plants inoculated with *Fusarium oxysporum* f. sp. *luffae* isolate Fomh16; (**B**): grafted plants inoculated with *Fusarium oxysporum* f. sp. *momordicae* isolate Fomo33. The yellow arrow indicates the pathogen.

**Figure 4:**
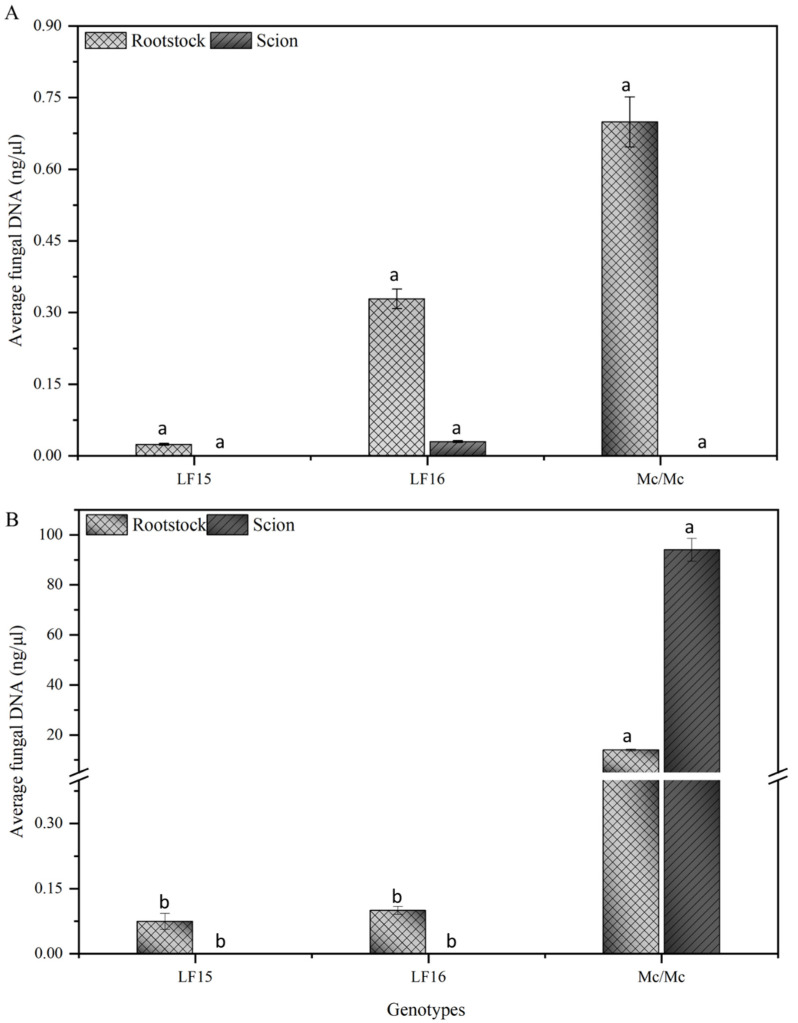
Quantity of the pathogen DNA in the luffa rootstock and bitter gourd scion at 28 dpi. (**A**): grafted plants inoculated with *Fusarium oxysporum* f. sp. *luffae* isolate Fomh16; (**B**): grafted plants inoculated with *Fusarium oxysporum* f. sp. *momordicae* isolate Fomo33. Error bars represent the standard deviation of triplicates. Different letters indicate significant differences between values according to Fisher’s protected least significant difference test (*p* = 0.05).

In this study, the colonization of the pathogen was monitored in the rootstocks and scions of grafted plants inoculated with both isolates Fomh16 and Fomo33. After inoculating the grafted plants with Fomh16 isolate, the pathogen can penetrate the roots and colonize the vascular bundle of all rootstocks by 28 dpi. However, the mycelium was restricted to the rootstock hypocotyl and did not extend to the bitter gourd scion (Figure 3). Isolate Fomo33 demonstrated the ability to infect and colonize the hypocotyl vascular bundle of all luffa rootstocks, except LF 15 and LF 16 rootstocks; the pathogen cannot be detected using microscopy observation. Notably, Fomo33 mycelium could only colonize and extend in the vascular tissue of bitter gourd scion grafted with rootstocks LF5 and LF11 at 28 dpi (Figure 4). Moreover, the pathogen was successfully re-isolated on PCNB media from all rootstocks of infected plants with both isolates. However, both isolates could only be recovered in minimal amounts from some plants of the LF15 and LF16 rootstocks and could not be recovered from most plants. In addition, the isolate Fomh16 cannot reisolate from all bitter gourd scion, while isolate Fomo33 was successfully re-isolated from the bitter gourd scion only when grafted onto LF5 and LF11 rootstocks (Table 3). After analyzing their morphological and molecular characteristics, all the fungal isolates obtained from grafted plants were identified as either Folu or Fomo.

### 2.4. Quantification of Fomh16 and Fomo33 Isolates in Grafted Plants

Real-time PCR was used to determine the quantity of DNA of Fomh16 and fomo33 in luffa rootstocks and bitter gourd scions at 28 dpi. The results of a standard curve revealed that the primers Fol02 and Fol03 produced target peaks with a melting temperature of 82 °C. A linear correlation was observed between the concentration of Fomh16 gDNA and real-time quantification cycles (*R*^2^ = 0.996) with efficiency rates (E > 101.7%) (Figure S1). The primers Fom13 and Fom14 produced target peaks with a melting temperature of 79.5 °C. A linear correlation was observed between the concentration of Fomo33 gDNA and real-time quantification cycles (*R*^2^ = 0.999) with efficiency rates (E > 102%) (Figure S1). Both primers achieved high efficiency in detecting DNA concentrations with the five different amounts of Fomh16 and Fomo33 gDNA.

The quantification of the pathogens in the plant samples indicates significant differences in the amount of the Fomo33 DNA between the bitter gourd grafted onto luffa hybrids and the self-grafted plant *Mc/Mc*. The Fomo33 DNA quantity in the LF15 and LF16 rootstocks were 0.074 ng/μL and 0.099 ng/μL of total plant DNA, respectively, at 28 dpi; however, the pathogen cannot be detected in the bitter gourd scions. In the self-grafted plants *Mc/Mc*, the amount of Fomo33 DNA was exceptionally high at 14 ng/μL of total plant DNA in the rootstock and 94 ng/μL of total plant DNA in the scion at 28 dpi (Figure 4). In contrast, no significant differences were detected in the amount of the Fomh16 DNA between the bitter gourd grafted onto luffa hybrids and the self-grafted plant *Mc/Mc*. The Fomh16 DNA quantity in the LF15 rootstocks measured 0.023 ng/μL of the total plant DNA, and none of the scion samples showed detectable Fomh16 DNA at 28 dpi. In bitter gourd plants grafted to LF16, the Fomh16 DNA was 0.32 ng/μL of the total plant DNA in the rootstock and 0.029 ng/μL of the total plant DNA in the scion. In the self-grafted plants *Mc/Mc*, the Fomh16 DNA amount was 0.69 ng/μL of the total plant DNA in the rootstock, and no pathogen DNA was detected in the scion (Figure 4).

## 3. Discussion

*Fusarium oxysporum* is a major plant pathogen responsible for vascular wilt and root rot in a variety of crops, including bitter gourd. Screening for Fusarium wilt resistance may provide a potential way for efficiently applying resistant plant materials to improve cultivar resistance [30]. Previous studies have identified resistant sources of Fusarium wilt in luffa, which could aid breeding programs aimed at developing new varieties or rootstocks to control soil-borne diseases in cucurbit crops [17]. In this study, nine luffa hybrids were obtained through crosses between four parental lines that displayed high resistant reactions to four isolates of Folu (Fomh16, FOLUSC, FOLUST, and Fol114), which were used as rootstocks for managing Fusarium wilt disease in bitter gourd.

Folu isolate Fomh16 and Fomo isolate Fomo33 were used in this study to evaluate luffa hybrids and bitter gourd grafted onto luffa rootstock. Both isolates were tested for their pathogenicity against the highly susceptible luffa and bitter gourd cultivars. Our results indicated that the Fomh16 isolate was highly pathogenic to the luffa but nonpathogenic to the bitter gourd, with no symptoms that occurred on the infected plants at 28 dpi. On the other hand, the Fomo33 isolate was highly pathogenic to the bitter gourd cultivar, while it did not show any pathogenicity to the luffa cultivar. Chung et al. [31] revealed that Fomh16 and Fomo33 isolates were highly pathogenic to luffa and bitter gourd plants. However, the Fomo33 isolate was reported to infect only the luffa open pollination cultivar and cause wilting symptoms [31]. The differences between the two sets of results could be attributed to using different luffa genotypes. Also, Namisy et al. [17] reported that luffa genotypes are an essential factor affecting the virulence of Folu. Moreover, certain factors, such as plant age and concentration of spore suspension, could influence disease severity in inoculation systems [32]. This study utilized a high concentration of spore suspension (1.5 × 10^6^ conidia mL^−1^ for 30 min) to ensure that only the most resistant plants could survive severe stress. This type of screening offers numerous advantages, including faster disease occurrence and a shorter assay period [33].

In the first evaluation, nine luffa hybrids were evaluated for their resistance to Fomh16 isolate; five hybrids (LF1, LF2, LF3, LF15, and LF16) showed resistance without displaying any disease symptoms. However, some plants within the resistant hybrid LF10 showed phenotypic variation; the severity of symptoms varied across the affected individuals, with the MDR ranging from 0.1 to 5. Namisy et al. [17] reported that among the resistant *L. aegyptiaca* (LA115, LA116, LA117, and LA122) and *L. acutangula* (LA142, LA143, LA144, and LA145) genotypes, few plants exhibited varied levels of phenotypic variation in response to one or more Folu isolates; these reactions ranged from a few symptoms to clearly susceptible reactions. The observed variation in some luffa hybrids in this study suggests that the resistance to Fusarium wilt could be quantitative and regulated by multiple gene combinations or environmental variations. Similar results have been reported in previous studies [32,34].

Grafting onto resistant rootstocks has effectively managed soil-borne diseases and root-knot nematodes in solanaceous and cucurbitaceous vegetables [22,35,36]. Moreover, this technique has also been reported to improve resistance to some foliar diseases and viruses in grafted plants, making it a valuable tool for disease management [22,24,37]. In this study, seven luffa intraspecific hybrids that showed resistance and moderate resistance to Fomh16 isolate in the first evaluation were used as rootstocks with bitter gourd (cv. Moon shine) to control Folu and Fomo. Based on the results, it was found that the bitter gourd plants grafted onto two rootstocks, LA15 and LA16, showed high resistance to both Fomh16 and Fomo33 isolates without exhibiting any wilting symptoms. Furthermore, three grafted rootstocks were resistant, with only a few wilting symptoms compared with self-grafting bitter gourd *Mc/Mc* and luffa *La/La*, which showed high susceptibility to Fomo33 and Fomh16, respectively. These findings indicated that luffa rootstock could effectively manage Fusarium wilt in bitter gourd. Previous studies have shown that luffa was utilized as a suitable rootstock in Asia to manage Fusarium wilt in bitter gourd [29]. Grafted watermelon onto *L. cylindrica* rootstock could successfully control races (0, 1, and 2) of *F. oxysporum* f. sp. *niveum* [38]. In addition to managing soil-borne disease, luffa rootstock can improve abiotic tolerance, such as drought and salt stress [26,27]. Most available rootstocks were obtained by screening germplasm, and very few were developed by crosses. However, breeding resistant hybrid rootstock genotypes is increasingly popular in the private sector [23]. Breeding programs can efficiently target above- and belowground traits and combine horticultural characteristics and disease resistance into a graft hybrid [39]. In this study, we used luffa intraspecific hybrid as rootstock with bitter gourd. Hybrid rootstock can produce a robust root system, leading to healthy shoot growth, stable resistance against diseases, and increased yield production. Intraspecific and interspecific hybrid rootstocks can improve resistance to soil-borne diseases in tomatoes, watermelons, and melons [21,36,40]. However, the interspecific hybrid rootstocks may reduce fruit quality [21], for instance, the interspecific hybrid rootstock, ‘Shin-tosa’ [*Cucurbita maxima* (Duchense ex. Lam.) × *C. moschata*], is resistant to Fusarium wilt in melon; however, it causes reduced fruit quality, including low sugar content, and fibrous flesh [41].

The survival rate of grafting is crucial for the success of grafted plants. It is influenced by various factors, including external environmental conditions, the grafting technique, and the compatibility between rootstock and scion [42]. In this study, our results indicated that bitter gourd scion grafted with all resistant luffa hybrids displayed a high survival rate at 28 dpi with both isolates, Fomh16 and Fomo33. It is suggested that the combination of bitter gourd and resistant luffa rootstocks is highly compatible. The grafted combination with good compatibility was reported to survive, grow more vigorously, and improve fruit quality and yield production [43]. Guo et al. [26] reported that luffa rootstocks grafted with cucumber showed high graft compatibility, enhanced salt tolerance, and improved quality and yield of grafted cucumber plants. Moreover, in grafted watermelon, the survival rate, yield, and quality of compatible combinations were significantly higher than in incompatible grafting [38].

*F. oxysporum* has been reported to penetrate the roots and invade the vascular system during the infection, causing wilting symptoms in cucurbits crops, including cucumber, melon, and watermelon [44,45,46,47]. In susceptible plants, the pathogen could colonize the vascular tissue and block the nutrient transfer, causing wilting; in contrast, the resistant plant could detect the pathogen early, activate a defense response, and restrict the pathogen in the cortex [48]. Nevertheless, some resistant genotypes may display slight symptoms [17]. Here, we observed that isolate Fomh16 could colonize the vascular tissues of all resistant luffa rootstocks; however, the pathogen was restricted in the rootstock base and could not extend and colonize the vascular tissues of bitter gourd scion. In addition, the isolate Fomo33 could colonize the vascular bundle of most luffa rootstocks but only extend and colonize bitter gourd scion grafted with LA5 and LA11, which displayed moderate resistance and susceptibility, respectively. Plants’ defense against pathogen attack includes preventing or restricting pathogen invasions or utilizing systemic defense mechanisms; grafting onto resistant rootstock may increase systemic defense [22]. Although both isolates Fomh16 and Fomo33 could colonize the luffa rootstock, only a few symptoms ranging from 0 MDR to 0.7 were displayed on the grafted plants. Folu isolates could colonize the hypocotyl of 14 resistant accessions in luffa germplasm without displaying wilt symptoms at 28 dpi [17]. Further research revealed that Folu colonized the hypocotyl vascular tissue of resistant luffa genotypes but was reduced over time [49]. These results suggest that the resistant mechanism may be due to the formation of tyloses that block or restrict the movement of the pathogen [50] or may also be due to the accumulation of some secondary metabolites in xylem sap, such as peroxidases and phenols, which contribute to plant defense [12,51,52]. In addition, the crude aqueous extracts from the resistant luffa genotype showed antimicrobial activity and could inhibit Folu spore germination [49].

In this study, real-time PCR was used to examine the amount of Fomo33 and Fomh16 isolates in both rootstock and scion of the bitter gourd plants grafted with LF15 and LF16 rootstock. Based on qPCR analysis, the Fomo33 could be detected with only a small amount in the LF15 and LF16 rootstocks; however, the pathogen cannot be detected in the bitter gourd scion at 28 dpi. Similarly, *F. oxysporum* isolates can be detected in the resistant bottle gourd and two interspecific hybrid squash rootstocks; however, the pathogen was restricted to the watermelon scion and suppressed wilt symptoms [53]. In addition, the phloem sap contains mobile proteins that move across the graft union between the rootstock and scion [54]. These proteins play a crucial role in the growth and development of plants by regulating essential processes, such as the formation of adventitious roots. They are also important in plant defense against various stresses [54,55]. Proteomic analysis in watermelon scions grafted onto bottle gourd rootstock under *F. oxysporum* f. sp. *niveum* (Fon) infection revealed an accumulation of 39 different proteins in watermelon scion leaves; 18% of these proteins are associated with defense and stress processes, indicating that they act as a positive regulator in defense responses triggered by Fon in grafted watermelon [35]. Also, *F. oxysporum* f. sp. *ciceri* can penetrate resistant chickpea plants. However, the pathogen distribution was restricted, providing evidence of an efficient defense mechanism [48]. In the Fomh16 isolate, a small amount of the pathogen DNA could be detected in the rootstock of LF15, LF16, and self-grafted plants *Mc/Mc*, and a small amount of the pathogen DNA can be detected in the bitter gourd scion grafted with LF16 rootstock. However, the pathogen could not be observed in the scion section under a microscope, nor could it be isolated on PCNB media. Additionally, no symptoms were observed up to 28 days post-inoculation (dpi). Similarly, Chung et al. [31] reported that the Folu isolates Fomh16, FOLUST, and Fol114 can colonize the different parts of grafted bitter gourd plants without experiencing any wilting symptoms.

## 4. Materials and Methods

### 4.1. Plant Material and Grafting Procedure

Nine luffa hybrids obtained from crosses between four genotypes belonging to *Luffa acutangula* LA140, LA145, LA39, and LA44 were used in this study. In our previous study, these four genotypes showed various resistance levels to *F. oxysporum* f. sp. *luffae* isolates (FOLUST, FOLUSC, Fol114, and Fomh16). The seeds of these genotypes were obtained from the GenBank of the World Vegetable Center, Taiwan. Tow commercial cultivars luffa *La* (cv. Shimmery) belonging to *L. aegyptiaca* and bitter gourd *Mc* (cv. Moon shine) belonging to *M. charantia* were obtained from Known-You Seed Co., Ltd. (Kaohsiung, Taiwan) and used as susceptible controls. Before sowing, the rootstock seeds were treated by cutting the seed coat opposite the embryo site, then soaked in SDW and incubated at 30 °C for one day to achieve uniform seed germination. The seeds were sown directly into 50-hole seedling trays containing an autoclaved peat moss and perlite soil mixture (3:1). The scion seeds were sown five days after the rootstock to match their hypocotyl diameter. When the scion and rootstocks were at the appropriate developmental stage (when both cotyledons and the first true leaf developed), the bitter gourd seedling was grafted onto luffa hybrids using the cleft grafting method. The grafted plants were placed in a healing chamber with a relative humidity of around 95% for seven days. Following this, the plants were moved to a greenhouse and kept at a temperature between 21 and 30 °C for another seven days until the junction was healed.

### 4.2. Fungal Isolates and Pathogenicity Test

The pathogenicity test was performed using two *F. oxysporum* formae speciales: Folu isolate Fomh16 and Fomo isolate Fomo33 against susceptible commercial cultivars luffa *La* (cv. Shimmery) and bitter gourd *Mc* (cv. Moon shine). Isolate Fomh16 was isolated from infected bitter gourd scions grafted onto a luffa rootstock and isolate Fomo33 was isolated from a bitter gourd wilted plant. Those isolates were identified as Folu and Fomo based on the morphological characteristics and molecular phylogeny analyses using the sequence of the intergenic spacer (IGS), elongation factor 1 alpha (*EF-1α*), and secreted in xylem 6 (*SIX6*) [31].

### 4.3. Inoculum Preparation and Inoculation Assay

The nine luffa hybrids were initially evaluated for their resistance to Fomh16 isolate. The conidia suspension was prepared from two weeks of fungal grown on potato dextrose agar (PDA) and incubated at 28 °C for two weeks, as described by Namisy [17]. In brief, the conidia suspension concentration was adjusted to 1.5 × 10^6^ conidia mL^−1^. The luffa hybrids were inoculated when the second true leaves appeared. The healthy plants were uprooted, and the roots were washed under gently flowing water. A third of the root tips were then trimmed off and submerged in the conidial suspension for 30 min. The roots of mock plants were dipped in SDW. Inoculated and mock plants were planted in 3-inch pots filled with autoclaved peat moss and perlite soil mixture in a 3:1. The plants were placed in the glasshouse with temperatures ranging between 25 and 30 °C and between 80 and 85% humidity. The experiment used a randomized complete block design with three replicates and five plants per replicate. Five uninoculated seedlings of each accession were used as mock controls. The plants were irrigated daily and fertilized once weekly with a balanced proportion of nitrogen, phosphorus, and potassium (20%-20%-20%).

Only the luffa hybrids that showed resistance and were moderately resistant to isolate Fomh16 were utilized as rootstocks with bitter gourd and reevaluated for their resistance to Fomh16 and Fomo33 isolates. The inoculation method and growth conditions were similar as described above. The self-grafted bitter gourd cultivar *Mc/Mc* and self-grafted luffa *La/La* were used as a susceptible control to Fomo33 and Fomh16, respectively.

### 4.4. Plant Assessed

The inoculated plants were assessed once weekly for four weeks after inoculation. The severity of the disease was evaluated using a 0 to 5 rating scale, with 0 for no apparent disease symptoms, 1 for yellowing in the cotyledon leaves, 2 for the yellowing or wilting of one true leaf, 3 for either the wilting of two true leaves or plant stunting, 4 for the wilting of the majority of plant leaves, and 5 for plant death [17]. The MDR was calculated at 28 dpi according to this formula, MDR = Σ(N*_i_* × *i*)/N*_t_,* where N*_i_* refers to the number of plants that received each disease rating score *i* and N*_t_* refer to the total number of plants. These data were used to calculate the area under the disease progression curve (AUDPC) [56] using the following formula, AUDPC = Σ[(x*_i_* + x*_i_*_+1_) × (t*_i+_*_1_ − t*_i_*)/2]*,* where x*_i_* is the estimated disease severity at time t*_i_*, x*_i+_*_1_ is the estimated disease severity at time t*_i+_*_1_, and t*_i+_*_1_ − t*_i_* is the interval between the scoring dates *i* and *i_+_*_1_. Based on the MDR results, the plants were categorized as either resistant or susceptible to both isolates. Accessions with MDR ≤ 1 were resistant, 1 ≤ 2 were moderately resistant, 2 ≤ 3 moderately susceptible, and >3 were considered susceptible. The survival rate of grafted plants was calculated every seven days for 28 dpi. The survival rate was expressed as a percentage of the total number of grafted plants. The plants with wilting scion with MDR ≥ 3 were considered non-survival grafted.

### 4.5. Pathogen Recovery and Molecular Confirmation

To confirm the colonization of the pathogen in the rootstocks and scion, six infected grafted plants per accession were randomly collected at 28 dpi. Three non-inoculated plants per accession were used as mock controls. The scion and rootstock pieces were first disinfected with 70% ethanol for 1 min, followed by 1% NaOCl for 5 min. Then, they were rinsed three times with SDW. The pieces were cut into approximately 0.5 cm sections under sterile conditions and placed on pentachloronitrobenzene (PCNB) medium from Sigma-Aldrich Chemie GmbH, Schnelldorf, Germany, supplemented with 200 mg/L of streptomycin and 100 mg/L of neomycin. The plates were examined four days after incubating at 28 °C in a dark. The fungal isolates were purified and identified as Fomo33 or Fomh16 through morphological and molecular analyses using two primers, fp7320/fp7337 and fp7319/fp7336, to amplify Fomh16 and Fomo33 DNAs, respectively [57].

### 4.6. Biomass Quantification of Fomh16 and Fomo33 Isolates in Grafted Plants

The primers Fol02 and Fol03 [49], along with Fom13 (5′-ACGTGCAAGCAGGGATACTT-3′) and Fom14 (5′-CCAAGATGGTGGGGAAACGA-3′) were utilized to quantify Fomh16 and Fom33, respectively, in the rootstocks and scions. The primers Fom13 and Fom14 were designed based on the sequences of *F. oxysporum* f. sp. *momordica* primers fp7319 and fp7336 [57] using Primer3Plus software v2.5.0 “https://www.bioinformatics.nl/cgi-bin/primer3plus/primer3plus.cgi (accessed on 13 June 2024)”.

A standard curve was generated by performing 10-fold serial dilutions ranging from 2.5 pg/μL to 25 ng/μL of Fomh16 gDNA and 10 pg/μL to 100 ng/μL of Fomo33 gDNA. The concentrations were adjusted using EzDrop 1000 (Blue-Ray Biotech, New Taipei City, Taiwan). The PCR reaction consisted of 10 μL of KAPA SYBR^®^ FAST Master Mix (Kapa Biosystems, Cape Town, South Africa), 0.4 μL of each primer, 7.2 μL of RNase-free ddH2O, and 2 μL of DNA template. The thermocycling conditions were as follows: 95 °C for 2 min, followed by 40 cycles of 95 °C for 15 s and 60 °C for 1 min. The samples were amplified in three biological replicates, and the standard curves were generated using the quantified target DNA and Ct values of the serial dilutions. The plant tissues were ground with liquid nitrogen. Then, the DNA was extracted from 200 mg of plant samples using the Plant Genomic DNA Purification Kit from (Genemark Technology Co., Ltd. in Tainan, Taiwan). The qPCR was carried out with CFX 384 Real-Time PCR System CFX 384 (Bio-Rad Laboratories, Hercules, CA, USA). The concentration of each DNA sample was adjusted at 25 ng amplification; non-template samples were used as a negative control, and the PCR reaction and the qPCR condition were adjusted as described above. The DNA amount of Fomh16 and Fomo33 isolates in the rootstocks and scions was estimated by comparing them to the standard curve.

### 4.7. Microscopic Examinations

The colonization of isolates Fomh16 and Fomo33 in the rootstocks and scion was observed at 28 dpi using Calcofluor White solution (Calcofluor White stain, Sigma, St. Louis, MO, USA) as described by Flores-Félix [58] with some modification. Three grafted plants from each genotype were selected and gently rinsed with tap water to remove soil particles during each sampling. Cross sections were prepared using an electro-freeze machine MA-101 (Komatsu Electronics, Tokyo, Japan). The sections were placed directly on glass slides and stained with 10 µL of Calcofluor White solution and 10 µL of 10% potassium hydroxide solution to improve the resolution. After that, the slide was covered with a glass cover and incubated at room temperature in the dark for 1 min for microscopy observations. The colonization of the pathogen on the vascular system of rootstock (1 cm below grafting point) and scion (1–2 cm above grafting point) was observed using the Axio Imager.A1 microscope equipped (Carl Zeiss AG, Jena, Germany) with a fluorescence illuminator system X-Cite 120Q (Excelitas Technologies, Pittsburgh, PA, USA) and Axiocam 506 color. The DAPI filter was used to visualize the fluorescence expression. The experiment was performed in triplicate and repeated twice, and about 30 slices per replication of each plant part were observed.

### 4.8. Data Analysis

Data analyses were conducted using the R Statistical Software v4.3.3 (https://www.R-project.org/). Data from the AUDPC and qPCR were analyzed using a one-way analysis of variance and Fisher’s least significant difference multiple comparisons test at *p* < 0.05 level. The standard errors of the means were also computed. To analyze the statistical significance of the grafted accessions on disease rating in each experiment, we used non-parametric Kruskal–Wallis analysis and Dunn tests for multiple comparisons (*p <* 0.05). To evaluate differences among luffa rootstocks on seedling survival rates over time, we employed Cox proportional hazard models for analysis and Kaplan–Meier curves for data visualization [59].

## 5. Conclusions

This study highlights an effective strategy to manage Fusarium wilt in bitter gourd (*Momordica charantia* L.) caused by *F. oxysporum* f. sp. *momordicae* (Fomo) through the use of resistant luffa hybrids as rootstocks. The evaluated hybrids demonstrated varying resistance levels to the pathogenic isolates Fomh16 and Fomo33, with two hybrids, LF15 and LF16, exhibiting robust resistance with no symptoms occurring on inoculated plants. Resistant rootstocks prevented the spread of the pathogen to bitter gourd scions. In addition, the quantitative PCR findings reinforce the effectiveness of these hybrids, as the presence of pathogen DNA was significantly lower in grafted plants compared to self-grafted plants. Despite a small amount of the pathogen, DNA can be detected in the bitter gourd scion grafted onto LF16 rootstock; the pathogen was not visible under the microscope in the scion section, nor could it be isolated on the PCNB medium. These findings contribute significantly to developing sustainable agricultural practices for disease management in bitter gourd cultivation. Moreover, these resistant materials could be important for further investigation of resistance mechanisms to Fusarium wilt in luffa plants.

## Figures and Tables

**Table 1:** Evaluation of luffa hybrids against *Fusarium oxysporum* f. sp. *luffae* isolate Fomh16 at 28 days post inoculation.

Rootstock Code	Luffa Species	Fomh16		
		MDR ^x^	AUDPC ^y^	Reaction ^z^
LF1	*L. acutangula*	0.0 ± 0.0 d	0.0 ± 0.0 c	R
LF2	*L. acutangula*	0.0 ± 0.0 d	0.0 ± 0.0 c	R
LF3	*L. acutangula*	0.0 ± 0.0 d	0.0 ± 0.0 c	R
LF5	*L. acutangula*	1.2 ± 0.24 c	20.5 ± 4.1 b	MR
LF6	*L. acutangula*	2.5 ± 0.26 b	45.9 ± 4.8 b	MS
LF10	*L. acutangula*	0.9 ± 0.05 cd	17.8 ± 0.9 bc	R
LF11	*L. acutangula*	1.6 ± 0.18 c	29.0 ± 3.1 b	MR
LF15	*L. acutangula*	0.0 ± 0.0 d	0.0 ± 0.0 c	R
LF16	*L. acutangula*	0.0 ± 0.0 d	0.0 ± 0.0 c	R
*La*	*L. aegyptiaca*	5.0 ± 0.0 a	87.5 ± 1.4 a	S

^x^ Mean disease rating (MDR) 28 days post inoculation; mean followed by ±standard error. Different letters in the same column indicate significant differences according to Dunn’s test (*p* = 0.05). ^y^ Mean value of area under the disease progression curve; mean ± standard error. Different letters in the same column indicate significant differences according to Fisher’s protected least significant difference test (*p* = 0.05). ^z^ Resistance category according to MDR 28 days post-inoculation. R: resistant (≤1); MR: moderately resistant (1 ≤ 2); MS: moderately susceptible (2 ≤ 3); S: susceptible (>3).

**Table 2:** Evaluation of bitter gourd plants grafted onto luffa hybrids for resistance against *Fusarium oxysporum* f. sp. *luffae* isolate Fomh16, and *Fusarium oxysporum* f. sp. *momordicae* isolate Fomo33 at 28 days post-inoculation.

Rootstock Code	Luffa Species	Fomh16			Fomo33		
		MDR ^x^	AUDPC ^y^	Reaction ^z^	MDR	AUDPC	Reaction
LF1	*L. acutangula*	0.4 ± 0.0 b	5.4 ± 1.5 cd	R	0.3 ± 0.3 bc	4.9 ± 4.9 c	R
LF2	*L. acutangula*	0.7 ± 0.7 b	11.4 ± 11.4 c	R	0.5 ± 0.3 bc	7.9 ± 4.0 c	R
LF3	*L. acutangula*	ND	ND	ND	0.4 ± 0.7 bc	5.4 ± 5.4 c	R
LF5	*L. acutangula*	1.6 ± 0.3 b	25.9 ± 5.2 b	MR	1.7 ± 0.1 b	17.3 ± 0.2 b	MR
LF11	*L. acutangula*	0.3 ± 0.3 b	4.7 ± 4.7 cd	R	3.8 ± 0.4 a	36.2 ± 3.1 a	S
LF15	*L. acutangula*	0.0 ± 0.0 b	0.0 ± 0.0 d	R	0.0 ± 0.0 c	0.0 ± 0.0 c	R
LF16	*L. acutangula*	0.0 ± 0.0 b	0.0 ± 0.0 d	R	0.0 ± 0.0 c	0.0 ± 0.0 c	R
*Mc/Mc*	*M. charantia*	0.0 ± 0.0 b	0.0 ± 0.0 d	R	4.3 ± 0.4 a	38.3 ± 4.9 a	S
*La/La*	*L. aegyptiaca*	5.0 ± 0.0 a	92.2 ± 0.0 a	S	0.0 ± 0.0 c	0.0 ± 0.0 c	R

^x^ Mean disease rating (MDR) at 28 days post-inoculation; means followed by ±standard error. Different letters in the same column indicate significant differences according to Dunn’s test (*p* = 0.05). ^y^ Value of area under the disease progression curve; means followed by ± standard error. Different letters in the same column indicate significant differences according to Fisher’s protected least significant difference test (*p* = 0.05). ^z^ Resistance category according to MDR at 28 days post-inoculation. R: resistant (≤1); MR: moderately resistant (1 ≤ 2); MS: moderately susceptible (2 ≤ 3); S: susceptible (>3). ND indicates a lack of data owing to the limited number of seeds or low germination rate.

**Table 3:** Re-isolate of *Fusarium oxysporum* f. sp. *luffae* isolate Fomh16, and *Fusarium oxysporum* f. sp. *momordicae* isolate Fomo33 from luffa rootstock and bitter gourd scion at 28 dpi.

Rootstock Code	Fomh16		Fomo33	
	Rootstock	Scion	Rootstock	Scion
LF1	+	−	+	−
LF2	+	−	+	−
LF3	+	−	+	−
LF5	+	−	+	+
LF11	+	−	+	+
LF15	+/−	−	+/−	−
LF16	+/−	−	+/−	−
*Mc/Mc*	+	−	+	+
*La/La*	+	+	+	−

The symbol “+” indicated that the pathogen could be re-isolated; the symbol “−” indicated that the pathogen could not be re-isolated; and the symbol “+/−” indicated that the pathogen could be re-isolated from some plants.

## Data Availability

All data generated or analyzed during this study are included in this published article.

## Supplementary Materials

The following supporting information can be downloaded at https://www.mdpi.com/article/10.3390/plants14081168/s1: Figure S1. Real-time PCR assay standard curve. A: *Fusarium oxysporum* f. sp. *luffae* isolate Fomh16 using primers pairs Fo102/Fo103; B: *Fusarium oxysporum* f. sp. *momordicae* isolate Fomo33 using primers pairs Fom13 and Fom14. A standard curve was generated using a 10-fold serial dilution. Each point represents the average of three biological replicates.
