# Supplementary figures and images for: Evaluation of Luffa Rootstocks to Improve Resistance in Bitter Gourd (Momordica charantia L.) Against Fusarium Wilt
Source: Plants (Basel). 2025 Apr 9;14(8):1168. doi: 10.3390/plants14081168 (PMC12030507; doi:10.3390/plants14081168)

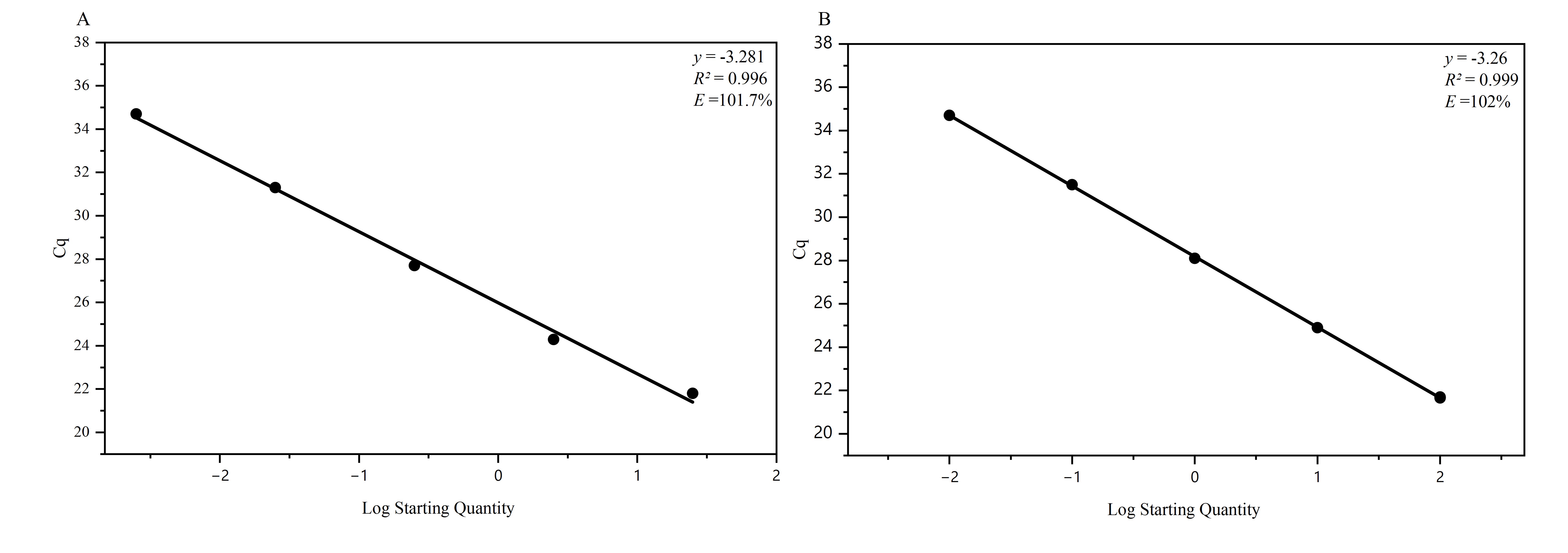

Supplement: Supplementary file 1 [file plants-14-01168-s001.zip › plants-3553056-supplementary.jpg]
